# Non-Alcoholic Fatty Liver Disease (NAFLD) and Its Connection with Insulin Resistance, Dyslipidemia, Atherosclerosis and Coronary Heart Disease

**DOI:** 10.3390/nu5051544

**Published:** 2013-05-10

**Authors:** Melania Gaggini, Mariangela Morelli, Emma Buzzigoli, Ralph A. DeFronzo, Elisabetta Bugianesi, Amalia Gastaldelli

**Affiliations:** 1 Institute of Clinical Physiology National Research Council, Pisa 56100, Italy; E-Mails: m.gaggini@ifc.cnr.it (M.G.); mariangela_morelli@yahoo.it (M.M.); emma.buzzigoli@ifc.cnr.it (E.B.); 2 Division of Diabetes, University of Texas Health Science Center, San Antonio, TX 78229, USA; E-Mail: albarado@iuthscsa.edu; 3 Department of Medical Sciences, Division of Gastro-Hepatology, San Giovanni Battista Hospital, University of Turin, Turin 10126, Italy; E-Mail: elisabetta.bugianesi@unito.it

**Keywords:** non-alcoholic fatty liver(NAFLD), steatosis, visceral fat, lipotoxicity, insulin resistance, free fatty acids, dyslipidemia, cardiometabolic risk

## Abstract

Non-alcoholic fatty liver disease is marked by hepatic fat accumulation not due to alcohol abuse. Several studies have demonstrated that NAFLD is associated with insulin resistance leading to a resistance in the antilipolytic effect of insulin in the adipose tissue with an increase of free fatty acids (FFAs). The increase of FFAs induces mitochondrial dysfunction and development of lipotoxicity. Moreover, in subjects with NAFLD, ectopic fat also accumulates as cardiac and pancreatic fat. In this review we analyzed the mechanisms that relate NAFLD with metabolic syndrome and dyslipidemia and its association with the development and progression of cardiovascular disease.

## 1. Introduction

**Figure 1:**
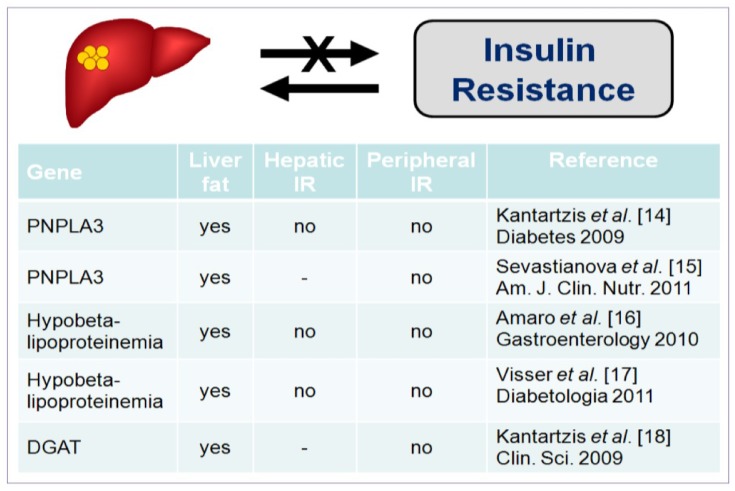
Non-alcoholic fatty liver disease (NAFLD) is not a primary determinant of insulin resistance, since subjects with hepatic TG accumulation due to genetic predisposition have a degree of hepatic and/or peripheral insulin resistance comparable to subjects without mutation.

Non-alcoholic fatty liver disease (NAFLD) has been considered a benign disease often associated with central obesity and insulin resistance and in general with factors of the metabolic syndrome (Figure 1). However, recent studies have highlighted that NAFLD is a chronic condition, ranging from benign steatosis, (*i.e.*, hepatic triglyceride accumulation >5.5% using magnetic resonance imaging [1,2] or >5% corresponding to 50 mg/g by wet weight), to more significant liver injury including lobular inflammation, hepatocyte ballooning, fibrosis and cirrhosis, *i.e.*, non-alcoholic steato-hepatitis (NASH) [3].

Excess liver fat is extremely common and prevalence of NAFLD has been increasing mainly because of the increased prevalence of obesity. It has been estimated that as many as 30% of adults in USA and other Western countries have NAFLD [4]. The real prevalence is unknown since NAFLD is often undiagnosed and most subjects with NAFLD, even those with diabetes, have normal liver aminotransferases and clinicians do not suspect the potential presence of NAFLD [5,6,7]. NAFLD is more prevalent among obese subjects and also in patients with type 2 diabetes independently of degree of obesity [7]. The prevalence increases to 57% in obese subjects, 70% in diabetic subjects and 90% in morbidly obese people. On the other hand NASH may be present in up to 3% of the general population and in up to two thirds of individuals with morbid obesity and/or type 2 diabetes [4,8]. In addition to hepatic complications, patients with NAFLD are at increased risk for cardio-metabolic complications such as type 2 diabetes (T2DM) and cardiovascular disease (CVD) [4,9].

In this article we have reviewed the current literature trying to highlight the mechanisms that are responsible for the development of NAFLD and that are at the base for the increased cardiometabolic risk in patients with NAFLD.

## 2. Insulin Resistance and the Development of NAFLD

Several studies have highlighted that insulin resistance is a characteristic feature of NAFLD [10,11,12], even when subjects are not obese [13]. However, NAFLD per se cannot be considered a cause for insulin resistance but rather a consequence as shown by studies in subjects genetically predisposed to NAFLD. In fact subjects with either mutation for PNPLA3 gene [14,15], familial hypobetalipoproteinemia [16,17] or mutation in DGAT [18,19], have fatty liver but peripheral and hepatic insulin sensitivity comparable to matched subjects without mutation and NAFLD (Figure 1). On the other hand NAFLD is highly prevalent among patients with type 2 diabetes (up to 70%) [20] that show increased hepatic triglyceride accumulation independently of BMI [7].

Insulin resistant subjects with NAFLD show reduced insulin sensitivity not only at the level of the muscle but also at the level of the liver and adipose tissue [7,13,21]. In insulin-resistant conditions, the adipose tissue becomes resistant to the antilipolytic effect of insulin and the release of fatty acids is increased [22]. Insulin resistance is accompanied by increased insulin levels that, in the presence of increased lipolysis and/or increased fat intake, promote hepatic triglyceride synthesis [7]. Adipose tissue insulin resistance is quantified using the index Adipo-IR (FFA × INS) [7,23] that reflects the inability of insulin to suppress peripheral lipolysis. In subjects with NAFLD, even if not obese, FFA concentrations and Adipo-IR are increased compared to control subjects [13,24], despite an increase in both hepatic and systemic lipid oxidation [13], and in VLDL-TG secretion [25,26]. Adipo-IR is also a marker of hepatic liver injury [21].

Under postprandial conditions, an important source of FFA is due to the increased spillover from chylomicrons [27]. The increased spillover reflects the inefficiency in dietary fat storage and results in excess FFA. FFA are taken up by organs saturating their oxidative capacity [13] and accumulated as ectopic fat, mainly as intramyocellular and hepatic lipids [28,29] but also as cardiac and pancreatic fat. It has been hypothesized that ectopic fat could be a defense mechanism against lipotoxicity [30,31] and that subjects with NAFLD develop NASH and cirrhosis only in consequence of a second hit due to increased inflammation and reactive oxygen species [3].

## 3. Impact of Hepatic and Visceral Fat Accumulation on Metabolic Profile

Hepatic (IH-TG) and visceral fat (VF) are highly correlated [7,32] but independently predict the presence of metabolic alterations [12,33]. The direct impact of VF on glucose and lipid metabolism is however controversial and difficult to prove since it is a small depot compared to subcutaneous fat [34]. Although a correlation is found between peripheral glucose clearance and IH-TG or VF [7,13,35], it is unlikely that IH-TG and VF contribute directly through FFA release and lipotoxicity to muscle insulin resistance. Although VF accounts for as much as 38% of total fat in some extreme cases [34], it is unlikely that FFAs released by VF are responsible for muscular lipotoxicity [36], except through cytokines released by the dysfunctional tissues.

**Figure 2:**
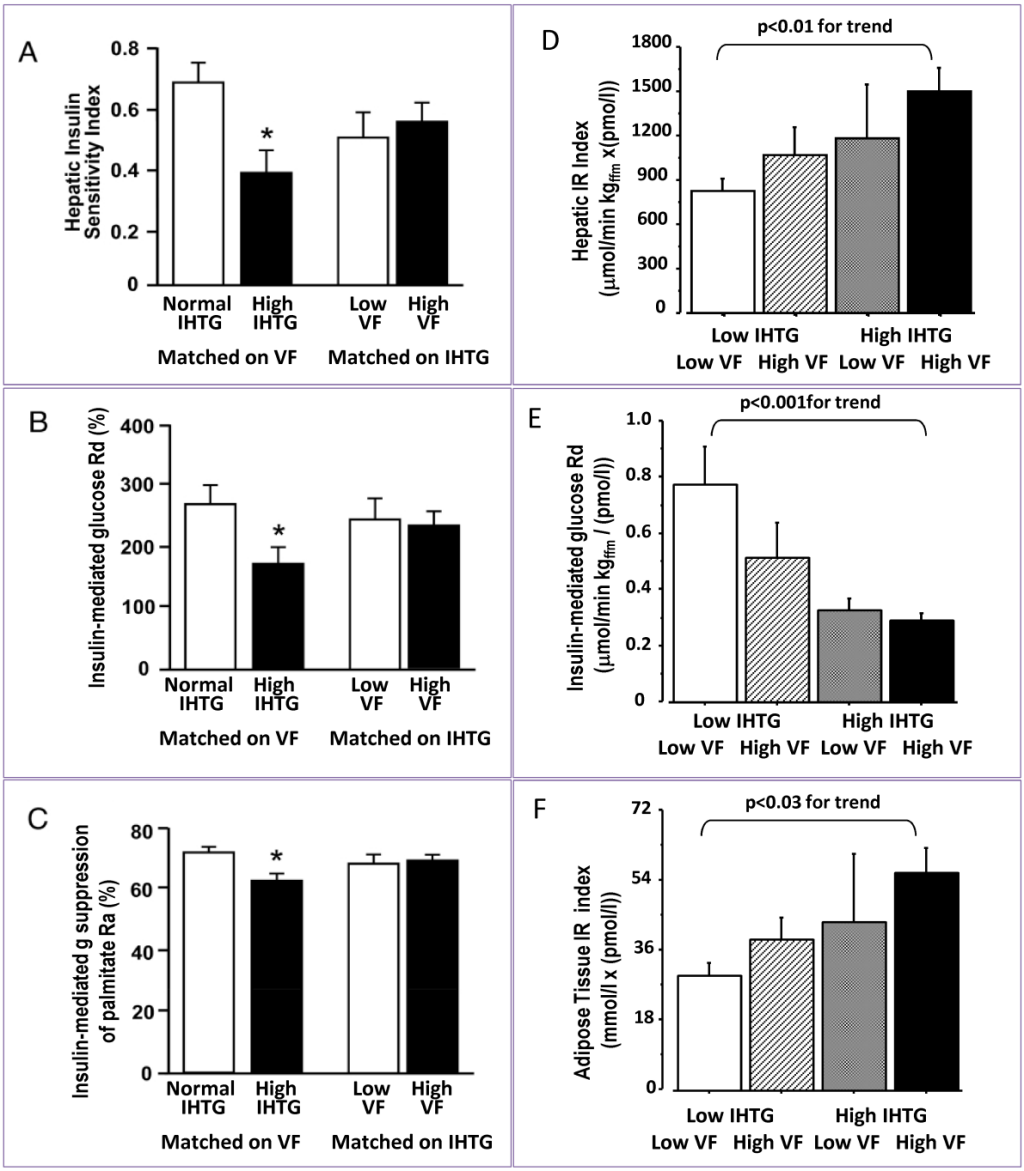
Right panel: Insulin resistance at the level of liver, muscle and adipose tissue is increased proportionally to both IH-TG and VF. Data redrawn from [7]. Average data for IH-TG and VF are shown in Table 1. Left panel: Subjects with similar but high VF (~1.3 kg) had increased insulin resistance if they had high IH-TG (~25%) compared to subjects with low IH-TG (4%) [12]. However, when matched per IH-TG (~13%), subjects with low and high VF had comparable indexes of insulin resistance (reproduced with permission from [12]).

**Figure 3:**
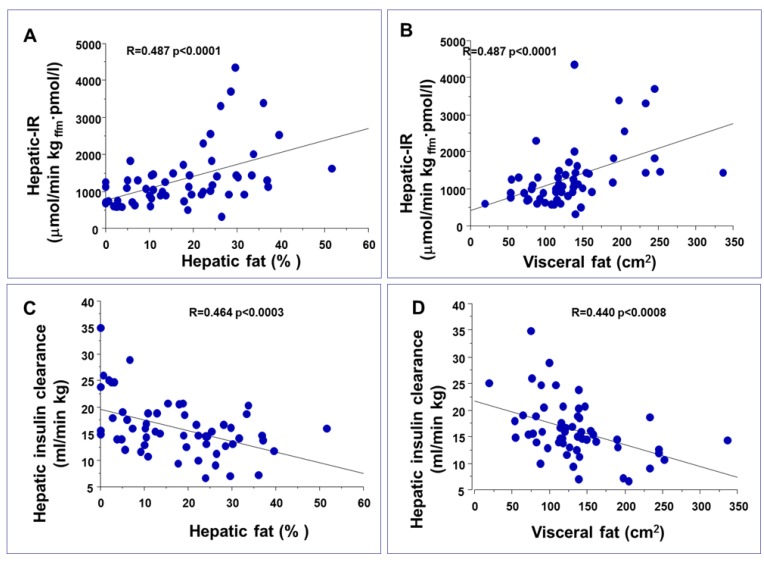
Similar correlations between liver and visceral fat accumulation, hepatic insulin resistance (*i.e*., the resistance to the effect of insulin to reduce fasting hepatic glucose production), top panels **A** and **B**, and between liver and visceral fat and hepatic insulin clearance, bottom panels **C** and **D**. Data redrawn from [7].

Previous studies indicate that hepatic fat and not VF was associated with insulin resistance. When subjects with different IH-TG content were matched on similar VF, they showed increased hepatic and peripheral insulin resistance and increased VLDL-TG secretion rate but no difference was observed between subjects matched for IH-TG but different VF [12] (Figure 2). Moreover, the partial VF reduction by omentectomy did not further improve peripheral and hepatic insulin sensitivity due to weight loss after surgery [37]. However, this type of analysis can be misleading, since subjects with normal IH-TG had high visceral fat content and subjects with matched IH-TG had on average 13% steatosis (Table 1). We therefore analyzed subjects with low VF and low IH-TG *vs.* those with both high VF and IH-TG, finding that insulin resistance increases proportionally to both visceral and liver fat (Figure 2D–F). By performing a simple correlation between hepatic and visceral fat and indexes of insulin resistance we observed that visceral and hepatic fat had a similar correlation [7] (Figure 3). 

**Table 1:** Hepatic and visceral fat in subjects matched for VF or IHTG (data from [12]) or when matched for low or high IHTG (data from [7]).

Group of subjects		IHTG (%)	VF	Reference
Matched on VAT	Normal IHTG	3.6 ± 0.5	1.29 ± 0.24 kg	[12]
	High IHTG	25.3 ± 3.5	1.34 ± 0.18 kg	
Matched on IHTG	Low VF	13.2 ± 3.5	0.76 ± 0.08 kg	[12]
	High VF	13.2 ± 3.3	1.94 ± 0.32 kg	
Low IH-TG	Low VF	2.1 ± 0.6	70 ± 8 cm^2^	[7]
	High VF	12.9 ± 1.9	84 ± 5 cm^2^	
High IH-TG	Low VF	3.2 ± 1.6	165 ± 41 cm^2^	[7]
	High VF	23.3 ± 1.7	159 ± 9 cm^2^	

The analysis reported in Figure 2, Figure 3 shows that IH-TG and VF contribute similarly to the increase in insulin resistance at all levels, liver, muscle and adipose tissue. In conditions of insulin resistance hepatic insulin sensitivity and hepatic insulin clearance are decreased proportionally to both hepatic and visceral fat content (Figure 3) and this is more evident in patients with Type 2 diabetes compared with non-diabetic subjects [7,35]. The relative contribution of IH-TG and VF to IR is in agreement with previous studies that have shown that obese subjects with increased VF have increased whole-body lipolysis compared to lean subjects [38,39] and subjects with NAFLD have preferential accumulation of abdominal fat. In upper body obesity, portal FFA concentrations, resulting from both systemic and VF lipolysis, is significantly greater than arterial FFA concentrations, exposing the liver to even greater amounts of FFA [40,41]. 

In the fasting state the liver is the main site of glucose production through gluconeogenesis (GNG) and glycogenolysis [42]. The contribution of GNG is increased in insulin resistant subjects [42] but because of increased circulating insulin, glycogenolysis is diminished and therefore hepatic glucose production remains within normal ranges, because of a mechanism named hepatic autoregulation [41,43]. Only when hepatic autoregulation is lost, *i.e.*, in T2DM, both GNG and glycogenolysis are increased and subjects develop hyperglycemia [42]. Subjects with NAFLD, despite high circulating insulin levels, have reduced hepatic insulin sensitivity and postprandial glucose clearance and increased free fatty acid and triglyceride concentrations [7,13,21,44]. Recently, Sunny *et al.* have shown an increase in GNG in a small group of subjects with NAFLD without known T2DM compared with controls [45]. We have found different results in a large group of T2DM patients: GNG was increased in T2DM *vs*. controls, but not associated with liver fat content [7]. On the other hand we have observed a direct relationship between GNG and VF and fasting hyperglycemia, while glycogenolysis was not correlated to either VF or IH-TG [7,46]. Liver fat in this study ranged from 0% to 52%, while in the paper by Sunny *et al*. [45] it ranged from 0% to 21% and this could explain at least in part the different results. Another difference could be the relative contribution of lipolysis and FFA to GNG. It is well established that elevated free fatty acids (FFA) stimulate hepatic GNG [47] and VLDL-TG production in the face of hyperinsulinemia [40]. Despite the differences in the type of NAFLD subjects (with or without T2DM) and the degree of liver steatosis, the lack of correlation between GNG and hepatic TG shows that not all subjects with NAFLD have increased GNG and indicates that hepatic TG do not participate directly to increase GNG. On the other hand the two pathways are independent and with different turnover rates, GNG is a dynamic process [48] while hepatic TG are not changing rapidly, e.g., after the intake of a high-fat meal change in hepatic TG was not significant [1]. Thus, we have hypothesized that in conditions of insulin resistance the increased lipolysis (especially from VF) generates an overload of glycerol and FFA to the liver that are “cleared” through increased TG synthesis and GNG (where glycerol is used as a gluconeogenic substrate and FFA provide the ATP necessary for the GNG process through hepatic beta-oxidation). Indeed, diabetic hyperglycemia is proportional to increased visceral fat content and GNG [46].

## 4. Fatty Liver, Dyslipidemia and the Metabolic Syndrome

**Figure 4:**
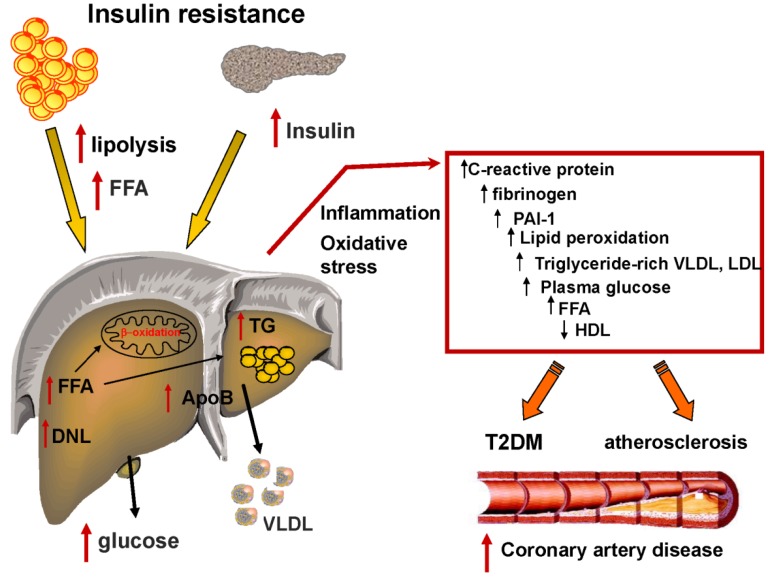
Link between insulin resistance and metabolic dyslipidemia. Insulin resistance is associated with an increase of free fatty acids (FFAs) flux that contributes to increased TG production that, in turn, stimulate assembly and secretion of VLDL in hepatocytes. Fat accumulation in the liver is associated with oxidative stress and lipid peroxidation. Furthermore NAFLD subjects have increased secretion of inflammatory markers, plasma glucose and a decrease in HDL concentration. The consequence of this physiological dysfunction is increased risk for the development of diabetes and atherosclerosis and increased risk for coronary artery disease.

The presence of dyslipidemia (hypercholesterolemia, hypertriglyceridemia, or both) has been reported in 20% to 80% of cases associated with NAFLD [49]. Liver fat content reflects the equilibrium between FFA flux through lipolysis, fatty acid oxidation, de-novo lipogenesis and VLDL secretion (Figure 4). The hepatic triglyceride accumulation is probably a consequence of saturation of fatty acid oxidation and VLDL secretion (Figure 4) since both these pathways are up-regulated rather than decreased in patients with NAFLD [13,25,26] and abnormality in apoB secretion has been excluded [25].

Postprandial hyperlipidemia and FFA spillover from chylomicrons worsen the situation [27,50]. Chylomicrons and triglyceride-rich lipoproteins can contribute either directly to plaque formation, following penetration of the arterial wall at sites of enhanced endothelial permeability, or potentially indirectly following liberation of lipolytic products (such as FFA and lysolecithin) which may activate pro-inflammatory signalling pathways in endothelial cells [51].

In patients with NAFLD adiponectin concentrations are decreased compared to normal subjects [52,53], despite higher lipolysis and fatty acid concentrations associated with increased hepatic and systemic fatty acid oxidation. It is well established that adiponectin activates AMPK and peroxisome proliferator-activated receptor (PPAR)-α, thus stimulating fatty acid oxidation in liver and muscle [54]. Adiponectin levels also correlate inversely with plasma TGs, positively with HDL-cholesterol levels and LDL size, suggesting a role in lipoprotein metabolism [26,55]. Thus, low adiponectin levels in NAFLD can be seen as a limit in the capacity of further increase lipid oxidation in response to FFA overload, redirecting FFA towards re-esterification.

### Fatty Liver and Metabolic Syndrome

It is not surprising that many studies have highlighted the association between NAFLD and several factors of metabolic syndrome, especially abdominal obesity, insulin resistance, increased serum triglycerides and small dense LDL and low HDL [7,25,26,44,56,57,58,59,60] (Figure 4). It has also been proposed that NAFLD could be considered the hepatic manifestation of metabolic syndrome [61,62]. Prevalence of the metabolic syndrome in NAFLD has been estimated to vary from 18% in normal-weight to 67% in obese subjects [61,62,63,64]. Moreover, liver fat accumulation is very common in type 2 diabetes [7] and a strong link has been observed between abdominal ectopic fat accumulation and the development of hypertension [62,65,66]. There are plenty of data linking the liver enzymes ALT and GGT (both correlated with liver fat) with incidence of diabetes [67]. It has been shown that up to 85% of subjects with NAFLD compared to 30% in controls are insulin resistant and have abnormal glucose metabolism, *i.e.*, prediabetes or T2DM, of which they were unaware [68]. This could explain the increased CVD risk that is often observed in patients with NAFLD highlighted by several epidemiological reports [9,69].

## 5. NAFLD and Cardiovascular Disease

NAFLD is associated with increased CVD [9,60,70]. The relationship between CVD and NAFLD (diagnosed with either ultrasound or liver biopsy) was evaluated in studies with limited number of subjects [67]. Thus, larger longitudinal studies are needed to demonstrate if NAFLD is a primary cause of CVD and/or if NAFLD per se increases the risk of CVD mortality. Several factors that can explain the increased CVD risk in subjects with NAFLD as already discussed in the previous paragraphs. Among these there are the increased lipolysis and VLDL secretion [13,25], the atherogenic lipoprotein profile, *i.e.*, increased small dense LDL fractions and reduced in HDL fractions [7,44,59,60], the hyperglycemia due to hepatic overproduction of glucose, the increased release of inflammatory factors such as fibrinogen and C reactive protein (CRP) [7,13,71]. Besides quantitative reduction in HDL concentration, also qualitative alterations occur [58], which may substantially contribute to the atherogenic risk of NAFLD.

Fat accumulation in the liver and oxidative stress induce the secretion of inflammatory markers such as, IL-6, TNF-α, Fetuin-A, CRP, and fibrinogen. Fetuin-A is a protein secreted by the liver, it is a natural inhibitor of the insulin receptor tyrosine kinase [72] and an endogenous ligand for TLR4 through which lipids induce insulin resistance [73]. It has been shown that Fetuin-A induces low grade inflammation [73,74], is associated with endothelial dysfunction [75], carotid atherosclerosis [76] and an increased risk of myocardial infarction (MI) and ischemic stroke (IS) [77] and type 2 diabetes [78]. Increased CRP promotes inflammation and accelerates atherosclerosis by increasing the expression of plasminogen activator inhibitor-1 and adhesion molecules in endothelial cells, inhibiting nitric oxide formation and increasing LDL uptake into macrophages [51]. All these metabolic abnormalities, common in subjects with NAFLD, have been shown to directly or indirectly promote atherosclerosis as confirmed by studies that showed increased carotid intima media thickness (IMT) and coronary atherosclerosis [9,44,60,69,71]. NAFLD is also associated with endothelial dysfunction and coronary artery disease [44,69,79,80,81].

The real prevalence of CV events in patients with NAFLD is still not known and probably underestimated. NAFLD is often not diagnosed since in the great majority of NAFLD subjects hepatic enzymes are within normal ranges and ultrasound technique is unable to detect NAFLD when fat infiltration is below 30% [82,83].

### 5.1. Fatty Liver and Atherosclerosis

Several studies have highlighted the association between NAFLD and increased carotid and coronary atherosclerosis [9,44,60]. In a large group of South Korean subjects, Sung *et al.* found that having fatty liver increases the risk of having coronary calcification and to develop type 2 diabetes [60]. In a Japanese cohort, age, obesity (body mass index BMI ≥ 25 kg·m^−2^), hypertriglyceridemia and, to a lesser extent, hypertension were among the variables that predicted development of fatty liver [84]. Previously it has been shown that hypertriglyceridemia is present in up to 64% [57] and indeed triglyceride and gamma glutamyl transferase (γGT) concentration, waist circumference and BMI are among the best predictors of fatty liver disease and related co-morbidities [44,85]. The RISC study showed that subjects with NAFLD are more prone to early carotid atherosclerosis even in the absence of metabolic syndrome and confounding diseases (hypertension, diabetes, cardiovascular diseases and dyslipidemia) [44]. The RISC study also documented the relationship between fatty liver and the presence of early plaques at carotid bifurcation, as well as the associations between carotid plaque presence and established atherosclerotic risk factors, family history of cardiovascular disease (FH-CVD) or diabetes, insulin sensitivity, serum liver enzymes, adipokines, free fatty acids and high-sensitivity C-reactive protein (hsCRP) [44,80]. 

### 5.2. Fatty Liver and Endothelial Dysfunction

Patients with NAFLD have endothelial dysfunction and a significant decrease in brachial artery endothelial flow-mediated vasodilatation compared to the healthy controls [20,79,86]. This decrease is correlated to histological features of NAFLD independent of age, sex, BMI, HOMA-insulin resistance, and other metabolic syndrome (MS) components [20,79]. The integrity and the maintenance of the endothelium wall are important in protecting against atherosclerotic vascular disease. The regeneration of the endothelial monolayer, when it is damaged, is performed by circulating bone marrow derived-endothelial progenitor cells (EPCs) and so the concentration of these in the plasma reflects the endothelial repair capacity [87]. Subjects with NAFLD have decreased plasma levels of EPCs that are correlated with arterial stiffening and endothelial dysfunction [88]. 

### 5.3. Fatty Liver and Coronary Artery Disease (CAD)

The presence of fatty liver is strongly associated with increased CAD risk [35,89] and CAD is a major cause of death in patients with NAFLD. This could also be explained by the fact that hepatic fat is often associated to cardiac fat and increased insulin resistance in these patients affects not only the liver but also other tissues like the heart [90]. The RISC study has shown that fatty liver is associated with an increased 10-year coronary heart disease risk score even in subjects without diabetes and hypertension, *i.e*., at low risk for CVD [44,80]. Moreover patients with NAFLD, even without metabolic syndrome, have more vulnerable coronary soft plaques than healthy controls [91]. In a large cohort of Taiwan workers, Lin *et al.* showed that patients with NAFLD were more likely to have CAD compared to patients without NAFLD, independent of obesity and other risk factors [89]. In patients with T2DM Targher *et al.* showed a higher prevalence of coronary, cerebrovascular, and peripheral vascular disease increased in those with NAFLD as compared to those without NAFLD [70]. However, despite the strong relationship between metabolic syndrome and CAD, it has been shown that some parameters of metabolic syndrome, like diabetes and hypertension, were better independent predictors of CAD than metabolic syndrome itself and that the association between NAFLD and CAD was independent of other demographic and metabolic factors [92].

## 6. Conclusions

In summary, NAFLD is associated with features of metabolic syndrome and is more prevalent among obese subjects and patients with type 2 diabetes independent of degree of obesity. The increased risk for cardio-metabolic diseases in NAFLD is caused by different factors among which hepatic overproduction of glucose, VLDL, inflammatory factors, C-reactive protein (CRP), and coagulation factors and by the presence of insulin resistance. Large trials that investigate the incidence of CVD and related mortality in subjects with NAFLD are needed to confirm this observation.
